# Leadership Development as a Medical Educational Fellow in Psychiatry: Reflection After Two Years

**DOI:** 10.1192/bjo.2024.307

**Published:** 2024-08-01

**Authors:** Jiann Lin Loo, Andrea Taylor-Clutton

**Affiliations:** Betsi Cadwaladr University Health Board, Wrexham, United Kingdom

## Abstract

**Aims:**

The disruptive force of the COVID pandemic has highlighted the importance of leadership for all medical educationists to prepare the future workforce with the ever-changing healthcare practice. Early career medical educators must attain leadership skills as early as possible. The only way to learn leadership is through experiential learning, i.e. learning while leading. Therefore, this self-study is aimed to share the reflection on the journey of a psychiatrist specialist trainee from North Wales in leading different psychiatric educational projects.

**Methods:**

This is self-study research on the reflective experience of working as a medical educational fellow while undergoing specialist training in psychiatry from January 2022 to December 2023. The data reflected were sourced from publications, end-of-project reports, meeting minutes, participant and peer feedback, personal records, educational portfolios, and appraisals.

**Results:**

Nine psychiatric educational quality improvement projects (QIPs) had been conceptualised and implemented, i.e. three series of mock exams (Special Preparation in CASC Exam), continuous coaching and mentoring in portfolio-based learning (Café of RCPsych Portfolio), continuous mentorship in academic writing (Mini North Wales-Academic and Research Clinic), continuous peer supervision in psychotherapy (Gogledd Cymru-Peer Supervision in Psychiatry), mock interview for job application, digitalisation of departmental induction, psychopathology training (3P: Psychopathology for Postgraduate Psychiatrists Trainee), and two international collaborative educational programmes (Perinatal Psychiatry Perinatal and Infant Psychiatry Educational Programme of Wales, Tanzania, and Malaysia; and Bhutan Old Age Psychiatry Educational programme). Five peer-reviewed publications had been completed while the other academic writings were ongoing. Three of the projects (33.3%) were expanded from a Welsh initiative to the whole United Kingdom and a bigger team was formed to ensure sustainability could be achieved. Two projects (22.2%) started as an international collaborative project. All projects provided opportunities for the members of the QIP to obtain workplace-based assessments and evidence for yearly appraisal while improving the educational experience of trainees and professionals in the field of mental health.

**Conclusion:**

All challenges come with the opportunities to be innovative in problem-solving. Communication skills and people management are crucial for resource gathering and conflict resolution. Lastly, talent development is required as part of the effort to sustain all the projects.

